# Fatty acid tryptamide from cacao elongates *Drosophila melanogaster* lifespan with sirtuin-dependent heat shock protein expression

**DOI:** 10.1038/s41598-022-16471-1

**Published:** 2022-07-15

**Authors:** Kiko Kanno, Yasunari Kayashima, Kazuji Tamura, Takako Miyara, Kento Baba, Megumi Koganei, Midori Natsume, Shinjiro Imai

**Affiliations:** 1 School of Bioscience and Biotechnology, Tokyo University of Technology, 1404-1, Katakura, Hachioji, Tokyo 192-0982 Japan; 2 Department of Food and Nutrition, Yamanashi Gakuin Junior College, 2-4-5 Sakaori, Kofu-shi, Yamanashi 400-8575 Japan; 3 Meiji.Co., Ltd., 1-29-1, Nanakuni, Hachioji, Tokyo 192-0919 Japan; 4 Meiji Seika Pharma Co., Ltd., 788, Kayama, Odawara, Kanagawa 250-0852 Japan

**Keywords:** Biochemistry, Enzymes, Transferases

## Abstract

Life span is increasing in developed countries as Japan, and an aging society is becoming a problem. In fact, healthy lifespan is not extended, and it is desired to extend it by functional food. Cacao (*Theobroma cacao*) contains various active components and is considered a preventative agent against metabolic disease. In addition, it has long been thought that regular cacao intake extends a healthy lifespan. However, there is no direct evidence for this belief. The purpose of this study is to identify the cacao component that elongate the lifespan of *D. melanogaster* as a model organism and to elucidate its functional mechanism. The activation of sirtuins, a family of NAD+-dependent deacetylases, has been reported to extend the lifespans of various organisms. Heat shock factor 1 is known to be deacetylated by reaction with sirtuins, thereby inducing gene expression of various heat shock proteins by heat stress and effectively extending the lifespan of organisms. Therefore, we evaluated whether components in cacao activate sirtuins and extend the lifespan of *D. melanogaster*. In the process, we discovered the fatty acid tryptamide as a lifespan-elongating component of cacao. Therefore, we investigated whether the fatty acid tryptamide from cacao upregulates the genes of heat shock proteins. As a result, it was confirmed that the gene expression of multiple heat shock proteins was significantly increased. This suggests that fatty acid tryptamide may activate sirtuins, increase gene expression of heat shock proteins, and elongate the lifespan of *D. melanogaster*.

## Introduction

In the process of searching for functional foods that promote health and longevity, we have found that the alkylresorcinols (ARs) contained in rye and wheat accelerate the enzymatic activity of SIRT1 and exert a lifespan-extension effect for *D. melanogaster*^1^. ARs are phenolic lipids composed of long aliphatic chains and resorcinol-type phenolic rings and, they are found in numerous plant species. SIRT1 stands for sirtuin (silent mating type information regulation 2 homolog) 1 and, is a type of NAD+-dependent protein deacetylase located in the cell nucleus conserved from Escherichia coli to humans^2^ and contributes to various regulation of cells by deacetylation of transcription factors. Sirtuins play important roles in gene silencing, DNA repair^3^, rDNA recombination^4^, and ageing in model organisms^5–7^. In the silencing function, sirtuin deacetylates a specific lysine residue of histone, so that the chromosome has a heterochromatin structure and contributes to its stability^8^.

In diverse species, the lifespan is extended when calories in the diet are restricted, suggesting that there is a conserved mechanism for nutrient regulation associated with aging^9–11^. It is important that there is no difference in food intake between the sample and the control in assessing the lifespan, as calorie restriction can extend *D. melanogaster* lifespan.

On the other hand, cacao contains ingredients that are effective for various health functions, and is said to promote health and longevity. In fact, there are report that cacao confers lifespan extension in *D. melanogaster*^12^ and *Caenorhabditis elegans*^13^. The ingredient that promotes their longevity is thought to be polyphenols^12,13^. However, we considered that cacao components other than polyphenols may contribute to life extension, and hypothesized ARs as a candidate. Since ARs are contained in other grain plants such as wheat^14^, we assumed that cacao might also contain AR, or other AR-like content. We hypothesized that cacao extract might have similar functions to ARs and we examined whether cacao extract has a function related to that of sirtuin. We also investigated what target molecules undergo sirtuin-like histone deacetylation by the cacao extract.

Heat shock factor 1 (HSF1) is essential for protecting cells from protein-damaging stress associated with misfolded proteins and has also been found to regulate aging^15^. Human HSF1 is inducibly acetylated with important molecule that negatively regulate DNA-binding activity^15^. Activation of deacetylase and the longevity factor SIRT1 prolongs HSF1 binding to the heat shock promoter Hsp70 by maintaining HSF1 in a deacetylated state. Conversely, downregulation of SIRT1 accelerated heat shock response (HSR) attenuation and release of HSF1 from cognate promoter elements^15^. If cacao extract increases the deacetylation-catalyzed reaction of SIRT1, resulting in extended lifespan of *D. melanogaster*, HSF1 may be involved in its activity. Therefore, in the lifespan evaluation of *D. melanogaster*, comprehensive gene expression analysis was performed for genes induced by HSF1 such as heat shock protein. We sought to elucidate the mechanism of HSF1 involvement in the regulation of longevity and establish the role of SIRT1 in protein homeostasis and HSR. *D. melanogaster* is a genetically accessible model organism that is important for aging research^16^. On the other hand, in recent years, rodents such as mice have been avoided from being used in experiments from an ethical point of view. Another advantage of *D. melanogaster* is that it has a shorter lifespan than mice, and results can be obtained in a short period of time. For these reasons, *D. melanogaster* was used as a model animal in the in vivo experiment of this study.

## Results

### Purification and structure determination

**Figure 1:**
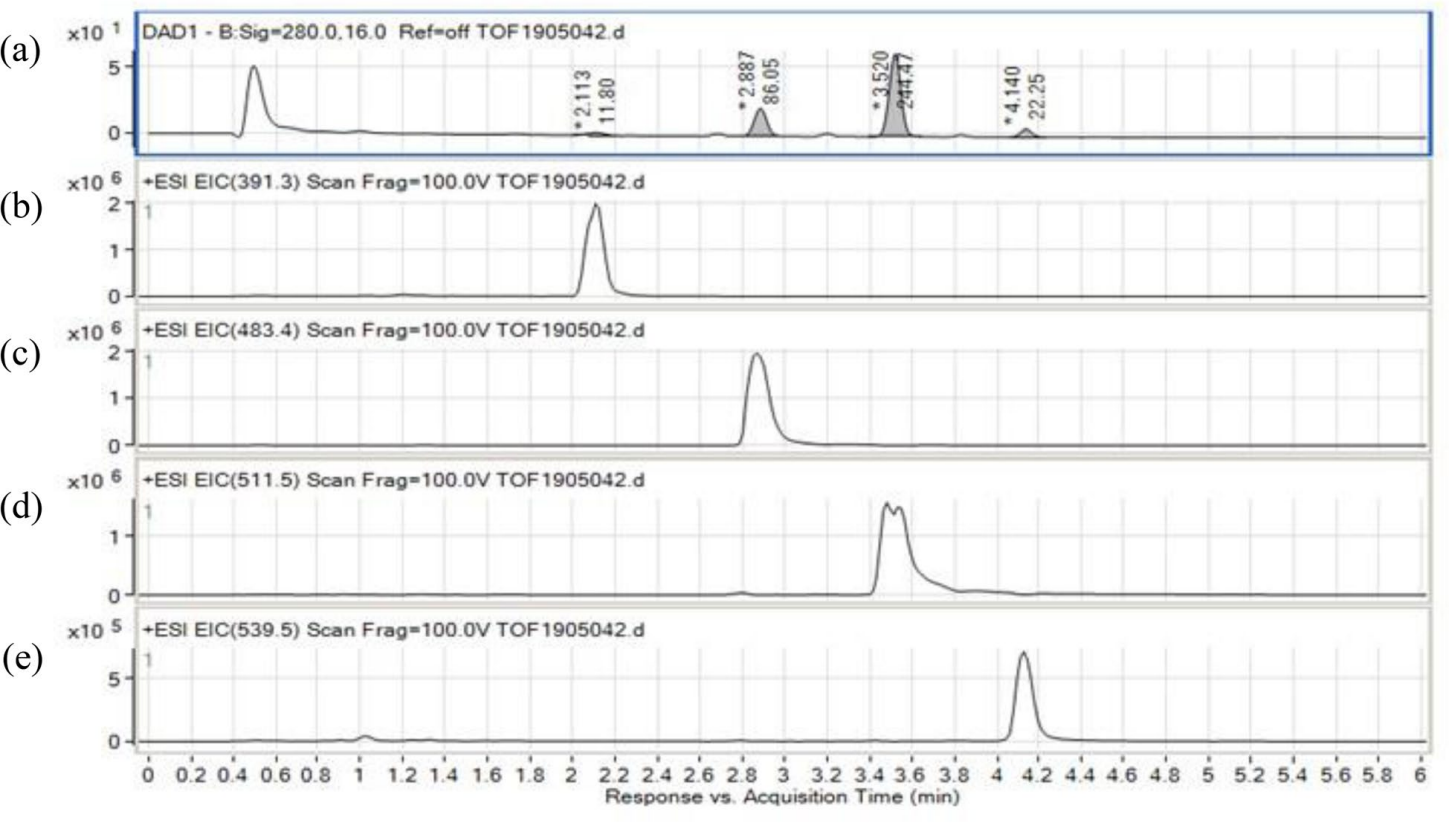
LC/MS result of the from 50 to 70 min fraction. (**a**) Chromatogram obtained at UV 280 nm. (**b**) Chromatogram of (M+H) +  = 391.3. (**c**) Chromatogram of (M+H) +  = 483.4. (**d**) Chromatogram of (M+H) +  = 511.5. (**e**) Chromatogram of (M+H) +  = 539.5.

**Figure 2:**
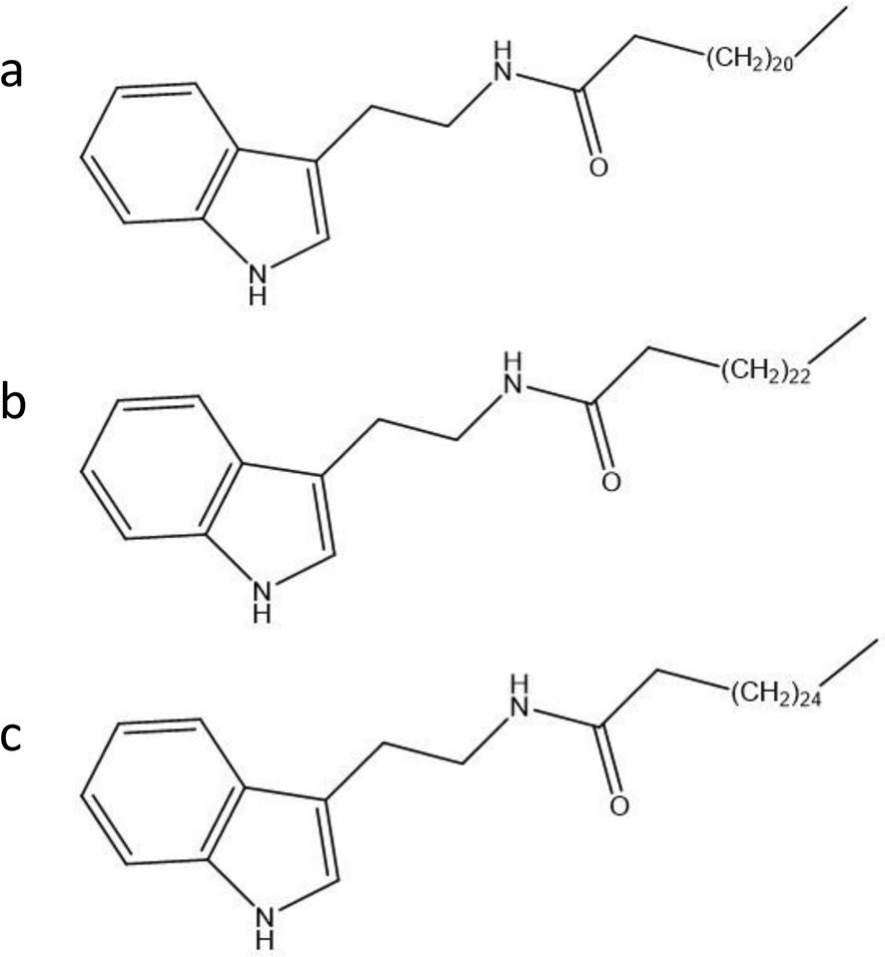
Search results of SciFinder. Docosanamide, N-[2-(1H-indo-yl)ethyl]- (**a**), Tetracosanamide, N-[2-(1H-indo-yl)ethyl]- (**b**) and Hexacosanamide, N-[2-(1H-indo-yl)ethyl]- (**c**) confirmed by SciFinder.

The results of LC–MS analysis confirmed a chromatogram peak at 50–70 min (Fig. 1). As a result of precise mass spectrometry, four compounds were separated (Table 1). Their molecular weight and structural formulas were estimated to be 390.2728 (C_24_H_38_O_4_), 482.4235 (C_32_H_54_N_2_O), 510.4547 (C_34_H_58_N_2_O) and 538.4868 (C_36_H_62_N_2_O), respectively. These peaks were isolated by HPLC and analyzed by GC/MS. As a result of GC/MS measurement and reference to the NIST library, peak 1 was estimated to be a phthalate ester and peaks 2, 3 and 4 to be compounds (fatty acid tryptamide) having an indole structure. When the latter three structures (Fig. 2) were investigated with SciFinder, it was confirmed that peak 2 (Fa-Trp21) include a docosanamide, peak 3(Fa-Trp23) include a tetracosanamide, and peak 4 (Fa-Trp25) include a hexacosanamide.

**Table 1:** Precise analysis of the 50–70 min fraction of the LC–MS analysis.

Peak	RT	Area	Area sum (%)	Positive (m/z)	Negative (m/z)	Chemical Formula	Calculated exact mass
1	2.1	12.39	3.32	391.3	n.d.	C_24_H_38_O_4_	–
2	2.8	88.35	23.67	483.4	481.4	C_32_H_54_N_2_O	482.4236
3	3.4	249.46	66.82	511.5	509.5	C_34_H_58_N_2_O	510.4549
4	4.1	23.13	6.20	539.5	537.5	C_36_H_62_N_2_O	538.4862

### Deacetylation evaluation

**Figure 3:**
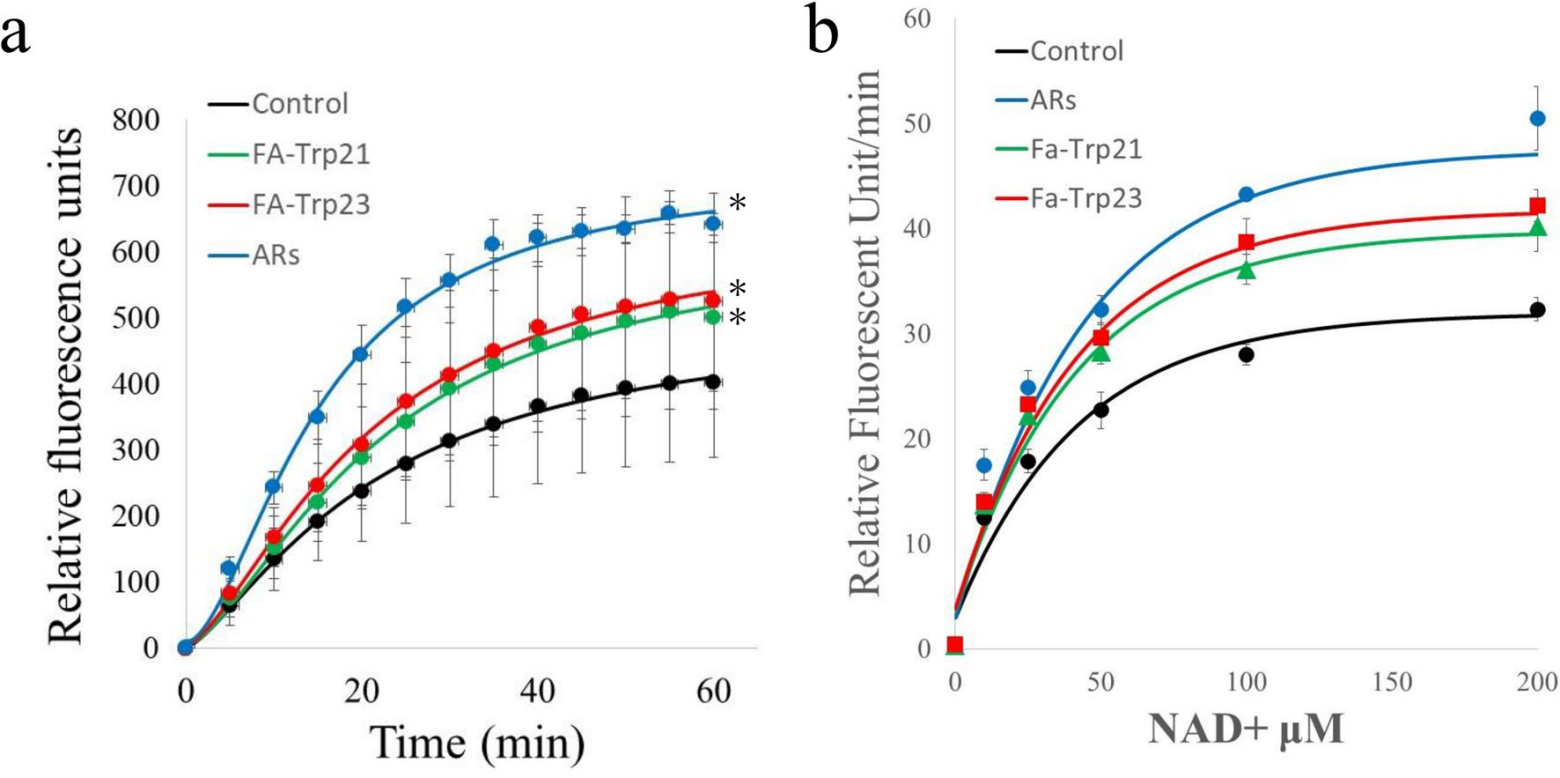
The effects of Fa-Trp on recombinant SIRT1. (**a**) The time course of SIRT1 deacetylation activity of Fa-Trp. Fa-Trp21, Fa-Trp23 and ARs were assessed at 10 μM with 100 μM of recombinant SIRT1 and 25 μM of NAD+. (**b**) Michaelis–Menten plot of Fa-Trp21, Fa-Trp23 and ARs for SIRT1 activity with various concentrations (12.5–200 μM) of NAD+ at 20 min. All values are the means of three determinations. Errors = s.e. *p < 0.05, compared with the control by Dunnett's method.

The results of the CycLex assay confirmed that Fa-Trp21, Fa-Trp23 and ARs as a positive control activated the deacetylation activity of SIRT1 (Fig. 3a). We plotted the results of our kinetic studies of SIRT1 activation for the control, Fa-Trp21, Fa-Trp23 and ARs (10 μM) as a Michaelis–Menten graph. When NAD+ was varied there no significant effect on the determination of the Michaelis constants (K_m_ = V_max_/2; control, 24.0 μM; Fa-Trp21, 24.2 μM; Fa-Trp23, 25.2 μM; AR, 25.6 μM), but there were pronounced differences in the apparent V_max_ (control: 78.1 unit/sec; Fa-Trp21, 88.4 unit/sec; Fa-Trp23, 92.7 unit/sec; AR, 114.9 unit/sec). Fa-Trp21, Fa-Trp23 and AR raised the V_max_ for NAD+ by 1.13-, 1.19- and 1.47-fold respectively (Fig. 3b).

**Figure 4:**
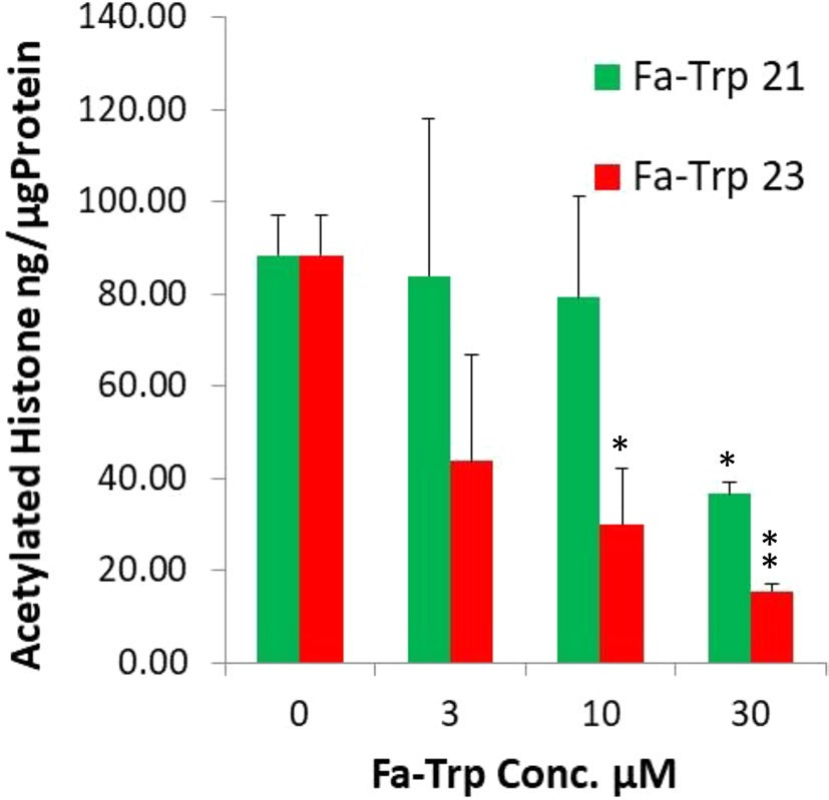
Effects of Fa-Trp on human monocyte cells for deacetylation of histone. Effects of Fa-Trp (10 μM) on TSA-insensitive deacetylase activity for histone in THP-1 cells. Errors = s.e. *p < 0.05, **p < 0.01 compared with the no added control by Dunnett's method. All values are means of at least three determinations.

To establish whether Fa-Trp can activate sirtuins in mammal cells, we performed a cell-based deacetylation assay using human cell lines. Unlike other classes of deacetylases, the sirtuins are insensitive to the inhibitor trichostatin A (TSA). Figure 4 shows the proportion of histones that were acetylated at each FA-Trp concentration. When 30 µM of FA-Trp21 was added, the proportion of acetylated histones decreased significantly compared with the control. When either 10 or 30 µM of FA-Trp23 were added to the THP-1 cells, the proportion of acetylated histones significantly decreased in a concentration-dependent manner compared with the controls.

### Evaluation of lipid metabolism regulation in mammalian cells

**Figure 5:**
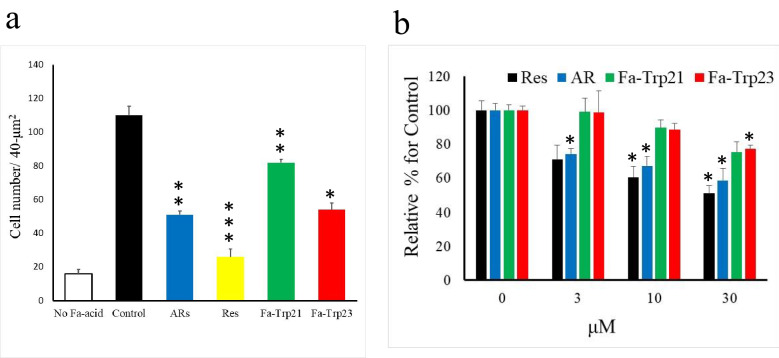
Effects of Fa-Trp lipid accumulation in HepG2 cells and 3T3-L1 adipocytes. (**a**) HepG2 cells were treated with 300 μmol/L palmitic acid conjugated by bovine serum albumin for 24 h. Oil Red O staining of HepG2 cells with Fa-Trp. The number of cells positive for Oil Red O staining was measured by taking a microscopic image of the cell culture and counting the number of cells stained red per 40-mm2 field. (**b**) Mouse 3T3-L1 cells were differentiated into adipocytes. After 24 h of stimulation with Fa-Trp, Oil Red O staining was performed. The relative optical density (OD) values of mature 3T3-L1 adipocytes were determined at OD540. All values are means of three determinations. Errors = s.e. *p < 0.05, **p < 0.01, ***p < 0.005, compared with the control by Dunnett's method.

To investigate the effect of Fa-Trp on fatty liver, fatty acids were added to the hepatoma cell line HepG2 culture medium, and the effect of Fa-Trp on fat-accumulated cells was evaluated. As shown in Fig. 5a, the number of Oil-red O-positive cells was significantly increased in cells to which palmitic acid had been added as a fatty acid compared with the untreated control cells. When palmitic acid-added cells were compared with cells treated by short-alkyl-chain alkylresorcinol olivetol and resveratrol as positive controls, the number of Oil-red O-positive cells was significantly lower for the positive controls. Furthermore, when Fa-Trp21 or Fa-Trp23 was added to cells, the number of Oil-red positive cells was significantly lower than that of the control.

The number of lipid droplets was increased by fatty acid treatment in Mouse 3T3-L1 cells induced to differentiate into adipocytes, as shown by Oil Red O staining. Fa-Trp, Res, and AR could reduce the lipid accumulation of adipocytes in a dose-dependent manner (Fig. 5b). However, the degree of decrease in lipid accumulation by Fa-Trp was milder than those of AR and Res.

### Dietary Fa-Trp in *D. melanogaster* adults

**Figure 6:**
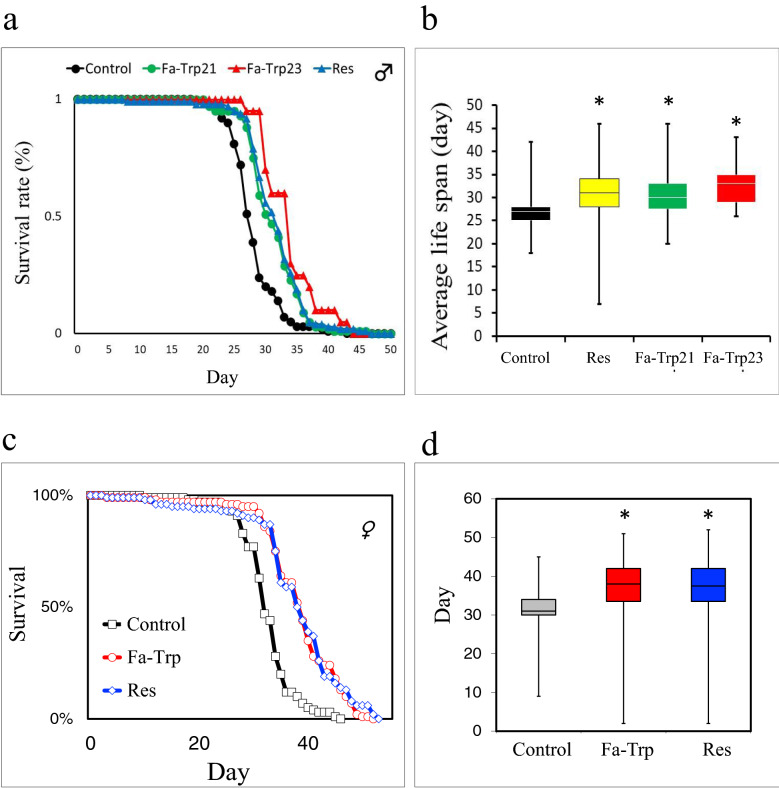
Dietary Fa-Trp induces longevity. (**a**) Survival curves by w1118 male flies reared on Fa-Trp21, Fa-Trp23, Res, and SF as controls. (**b**) Profiles for the lifespan of the male flies are shown as box plots. Dietary cacao derived Fa-Trp induces longevity in adult female *D. melanogaster*. (**c**) Survival curves by w1118 female flies reared on Fa-trp (SF + 0.07% cacao derived Fa-Trp, Res Control (Standard Food). (**d**) Profiles for the lifespan of the female flies are shown as box plots. Boxes and median lines represent the interquartile range and median values of data, vertical lines represent minimum and maximum data values. Sample sizes: n = 100 (Fa-Trp21, Res, control), 20 (Fa-Trp23). Errors = s.e. * p < 0.01, compared with the control by Wilcoxon signed-rank test.

The physiological effects of Fa-Trp on multicellular organisms was investigated using the fruit fly *D. melanogaster*. First, we investigated whether Fa-Trp affects the longevity of *D.* adults. As a result, adult male *D. melanogaster* fed with Fa-Trp in standard food (SF) showed a significantly prolonged mean survival rate compared to adult male *D. melanogaster* fed SF only (Fig. 6a,b). These findings suggest that ingested Fa-Trp may have increase the lifespan of adult *D. melanogaster*. Such mean survival extension was also shown in adult female *D. melanogaster* fed with cacao derived Fa-Trp in standard food (Fig. 6c,d). Since reduced food intake affects longevity, food intake was measured by the CAFE assay. As a result, there was no significant decrease in food intake in the Fa-Trp21, Fa-Trp23, and Res intake groups compared to the control group (data not shown).

**Figure 7:**
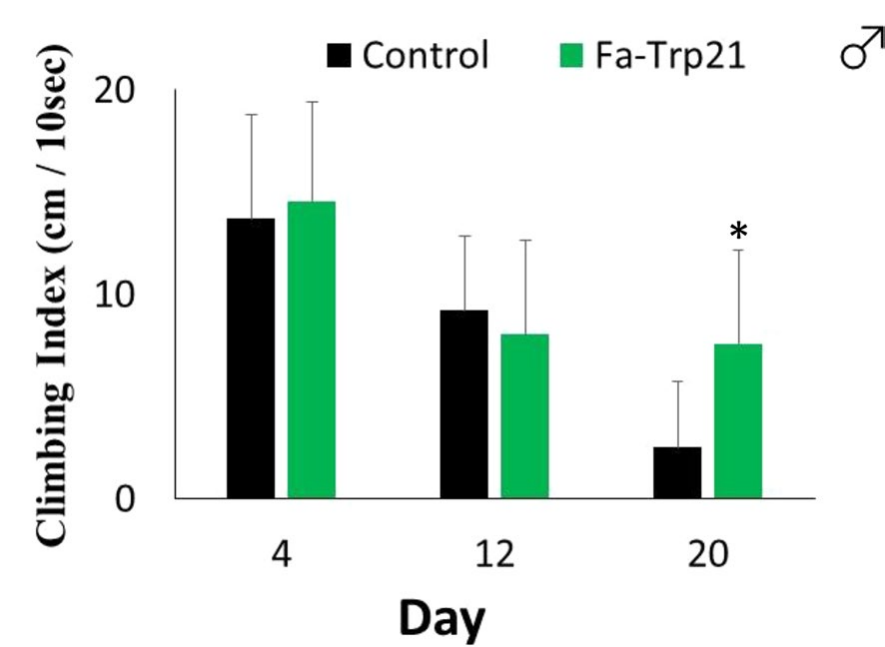
Fa-Trp ingestion suppresses individual senescence in adult *D. melanogaster*. Adult males with equal days post-eclosion were bred with SF as control or SF containing Fa-Trp, and a hill climbing assay was performed after 4, 12, and 20 days. Age-related muscle weakness seen in adults at 20 d post-eclosion was significantly suppressed in adults bred with SF containing Fa-Trp21 (*p < 0.01, compared with the control by Kaplan Meier method with Wilcoxon signed-rank test). N = 3 of 5 trials each.

A hill-climbing assay was performed to assess whether this lifespan extension was caused by senescence control. As a result, on the 20th day after eclosion, the insects bred on the standard food diet showed age-related muscle weakness, whereas the insects bred on the Fa-Trp diet had less muscle weakness (Fig. 7). Our findings demonstrate that Fa-Trp increases the lifespan of adult *D. melanogaster*.

We used RNA-seq analysis to screen for genes whose expression levels varied with Fa-Trp consumption and identified several genes whose expression levels fluctuate in a Fa-Trp dose-dependent manner. Gene set analysis using KEGG pathway data categorizing genes by function and related metabolic pathways revealed that only one of 113 groups showed variable expression of the longevity-regulating pathway in a Fa-Trp diet-specific manner. There are 54 genes in this longevity-regulating pathway. Among them, *Sirt1* significantly increased in the breeding group with Fa-Trp-containing baits (Table 2). Also, among the heat shock proteins regulated at the transcriptional level, genes with markedly increased expression were more prevalent in the rearing group with Fa-Trp-containing baits (Table 2).

**Table 2:** Alteration of the gene expressions of *Sirt1* and heat shock proteins in longevity regulating pathway in flies raised on Fa-Trp.

Symbol	Fold-change (vs.) control			
	Male		Female	
	Fa-Trp21	Fa-Trp23	Fa-Trp21	Fa-Trp23
*Sirt1*	*1.25	*1.42	*1.11	*1.44
*Hsc70-1*	1.06	0.90	0.97	1.04
*Hsc70-2*	0.93	1.56	1.03	0.90
*Hsp68*	*36.4	*91.2	*10.0	*41.4
*Hsp70Aa*	*8788	*10,205	*4.19	*13.8
*Hsp70Ab*	*21.1	*29.3	*42.7	*123.3
*Hsp70Ba*	*61.1	*116.2	*16.7	*112.8
*Hsp70Bb*	*42.3	*75.5	*13.5	*72.9
*Hsp70Bc*	*37.9	*65.0	*6.88	*32.5
*Hsp70Bbb*	*33.9	*72.7	*13.4	*75.4

## Discussion

Sirtuins are enzymes that catalyze NAD+ dependent protein deacetylation. The natural polyphenolic compound resveratrol received renewed interest when recent findings implicated resveratrol as a potent SIRT1 activator capable of mimicking the effects of calorie restriction^21^. The natural SIRT1 activators ARs increased the Vmax of recombinant SIRT1 for NAD+ and peptide substrate, and that ARs decreased acetylated histone in human monocyte cells by stimulating SIRT1-dependent deacetylation of substrates^1^. ARs also extended the lifespan of *D. melanogaster*, which was shown to be dependent on functional Sir2. We hypothesized that cacao include ARs as natural catalytic activators for sirtuin. We evaluated whether cacao nibs have histone deacetylation activity. Cacao nibs were shown to have histone deacetylation activity, and component analysis by high-performance liquid chromatography (HPLC) of cacao nibs confirmed the presence of components similar to ARs. However, when the HPLC peak was fractionated and structural analysis was performed by mass spectrometry, it was found that the key component was not an ARs but the fatty acid tryptamide (Fa-Trp)^17^, in which a fatty acid is amide-bonded to an indole structure. Although it has previously been reported that cacao contains the fatty acid tryptamide. Fatty acid tryptamide has only been reported to have the neurotrophic properties on dopaminergic (DA) cs in primary mesencephalic cultures^18^, with no other biological functionality reported.

Our experiments demonstrated that human SIRT1 deacetylase activities were increased with Fa-Trp diet supplementation. The results of our kinetic studies indicate that Fa-Trp increases the catalytic activity of SIRT1 by affecting the enzyme structure. This effect may be an allosteric SIRT1 activation by Fa-Trp, as in the case of ARs. We observed that Fa-Trp decreased the rate of TSA-insensitive histone acetylation in the human monocyte cell line THP-1. Unlike other classes of deacetylases, sirtuins are insensitive to the inhibitor TSA^19^. Since in this experiment, Fa-Trp has the activity of suppressing fat accumulation in HEP-G2 liver cancer cells, it may be effective in preventing fatty liver^20^. It is known that sirtuins’ role in catalyzing the deacetylation reaction promotes lipid metabolism and contributes to the burning of body fat^21^. Acceleration of deacetylation-catalyzed reactions by activation of sirtuins is believed to increase lipid burning and prevent metabolic diseases such as obesity. Resveratrol is also believed to accelerate deacetylation by sirtuin, increase lipid burning and prevent the development of various metabolic disorders^21^. However, resveratrol does not accelerate the deacetylation-catalyzed reaction, but prevents metabolic diseases by enhancing the gene expression of SIRT1^22^. Comparing Fa-Trp and resveratrol or alkylresorcinol, the results of the in vitro experiment in this study generally showed that Fa-Trp tended to be less active than resveratrol or alkylresorcinol. Alkylresorcinol activates sirtuins and accelerates deacetylation, but it showed no significant difference in activity compared to resveratrol. From these findings, it is considered that Fa-Trp’s relatively weak effect on lipid metabolism derives from its relatively weak acceleration of the deacetylation of sirtuins compared to alkylresorcinol. Although the effect of Fa-Trp on lipid metabolism is weaker than those of resveratrol and alkylresorcinol, Fa-Trp may still be effective in preventing metabolic diseases. In this experiment, Fa-Trp could reduce the lipid accumulation of Mouse 3T3-L1 adipocytes in a dose-dependent manner. 3T3-L1 adipocytes have some characteristics of brown adipocytes^23^, and Fa-Trp may cause brown adipocyte characteristics in 3T3-L1 adipocytes. Brown adipocytes contribute to maintaining body temperature in cold environments as special adipocytes that produce heat in response to cold exposure. The heat-producing and energy-consuming activities of these fat cells are expected to be useful not only for thermoregulatory ability but also for prevention of obesity and metabolic diseases^24^. Thus, Fa-Trp may be effective in controlling obesity.

Considering that the lifespan confirmation experiment in Sir2-deficient *D. melanogaster* cannot be carried out as described later, it cannot be concluded that the life extension of *D. melanogaster* by Fa-Trp is Sir2-dependent. However, considering the SIRT1 enzyme reaction rate-increasing activity by Fa-Trp, it is highly possible that Fa-Trp increases the lifespan of *D. melanogaster* in a Sir2-dependent manner. This effect may function through pathways associated with calorie restriction. If so, lifespan may be extended in the *D. melanogaster* experiment even when the Fa-Trp intake group consumes less food than the control group. However, there was no difference in food intake between the control group and the Fa-Trp intake group. Therefore, the results of this experiment show that the Fa-Trp extended the lifespan of *D. melanogaster*, not due to calorie restriction. The overexpression of sirtuins has been reported to increase the lifespan of *D. melanogaster*^25^. However, when the reported effects of sirtuin overexpression on aging were closely re-examined, it was found that standardization of the genetic background and the use of appropriate controls abolished the apparent effects in *D. melanogaster*^26^, because longevity depends on sex and genetic background^27^. In contrast, when an inducible gene switch system was used for the conditional expression of *Sirt1*, the *D. melanogaster* lifespan was increased^28^. The mild activation levels of sirtuin by Fa-Trp might extend the lifespan of *D. melanogaster*. The lifespan of a homeothermic animal varies greatly depending on the environmental temperature^29^. If the breeding temperature of *D. melanogaster* is lowered by 10℃, the average life span and maximum life span will be extended by about 2 to 3 times in the range of about 10 to 30 °C^30^. For this reason, our *D. melanogaster* survival experiments were conducted at 25 °C, but it is preferable to also conduct experiments at 15 °C or 37 °C. However, our *D. melanogaster* breeding environment facility cannot control breeding temperatures other than 25 °C. If experimented at 15 °C, the lifespan of *D. melanogaster* ingested Fa-Trp is expected to be longer than at 25 °C. We performed the longevity assay at 25 °C, but the optimal survival temperature for *D. melanogaster* was 15 °C, so it may be undeniable that a more significant difference could be seen at 15 °C. On the other hand, when experimented at 37 °C, the lifespan of *D. melanogaster* ingesting Fa-Trp may be shorter than at 25 °C due to the effects of heat shock. Since we have only conducted experiments in 25 °C breeding, we cannot conclude, but it seems that HSP is not induced by normal diet at least in 15 °C breeding. Since the induction of HSP expression by Fa-Trp is considered to be a response similar to that of heat shock, it can be assumed that HSP is induced even if a similar experiment is performed in a breeding at 15 °C, and as a result, the life span of *D. melanogaster* may extend. In addition, in order to confirm whether the life extension by Fa-Trp is Sir2-dependent, it is necessary to evaluate with Sir2-deficient *D. melanogaster*, but the Sir2-deficient *D. melanogaster* strain is eradicated at the stock center and is not available.

To characterize locomotor behavioral phenotypes for aging related genes, fly climbing assays have been widely used^31^. On the 20th day after eclosion of male fly, the insects bred on the standard food diet showed age-related muscle weakness, whereas the insects bred on the Fa-Trp diet had less muscle weakness. This result show that this lifespan extension was caused by senescence control. In female fly individuals, there is no significant change in motor performance throughout the adult period from our past experiment. Therefore, we did not that the Climbing Assay in female individuals.

It is known that the 80th lysine residue of heat shock factor 1 is deacetylated by the reaction of sirtuins and induces gene expression of various heat shock proteins by heat stress. Although it has not been confirmed whether Fa-Trp from cacao beans promotes the deacetylation of HSF1, we confirmed that the Fa-Trp from cacao induces the gene expression of heat shock proteins and demonstrated this gene expression.

It is known that increased *Sirt1* gene expression prolongs individual lifespans^32^. Heat shock proteins function as molecular chaperones^33^. They are usually expressed by external stresses such as heat and are responsible for protein folding. These our findings suggest that since ingesting Fa-Trp increased the *Sirt1* gene expression, the heat shock proteins are constantly expressed at a high level, and the physiological effect of proteins such as enzymes is to protect the higher-order structures that might have resulted in the inhibition of senescence and prolongation of lifespan. Epidemiological studies^34,35^ have indicated that cacao intake is protective against cardiovascular disease, diabetes, hypertension and aging. Sirtuins’ enzyme activities are critical regulators of these diseases and have primarily protective functions in the development of many age-related diseases^36^. Fa-Trp may have a central functional role in cacao due to its protection against these diseases.

In conclusion of this study, the fatty acid tryptamide activates sirtuins, increases gene expression of heat shock proteins and prolongs the lifespan of *D. melanogaster*. Activated sirtuins promote deacetylation of various proteins such as histones. The effect of fatty acid tryptamide on *D. melanogaster* may depend on inducing gene expression of heat shock protein by deacetylating HSF1.

FA-Trp is found in cacao nibs, but more abundant in the shell^37,38^. Therefore, continuous intake of chocolate made from cacao is effective in preventing these diseases. Most of the cacao shells are discarded because they are not used in chocolate production. Since cacao shell contains a large amount of Fa-Trp, effective use of cacao shell as a functional food is an issue for the future from the viewpoint of Sustainable Development Goals.

## Materials and methods

### Reagents

Docosanoic acid tryptamide (Fa-Trp21) and tetracosanoic acid tryptamide (Fa-Trp23) were purchased from Sigma-Aldrich (Milano, Italy).

### Extraction from cacao

According to the previous report, extraction was performed according to the following procedure^1^. Cacao nibs (300 g) from Ecuador were soaked in ethanol 6000 mL at room temperature overnight. Solvent was removed from the extract solution by rotary evaporator. The residue was extracted by ethyl acetate 1200 mL, followed by hexane 150 mL. The solution of this hexane extract was placed on a silica gel column (High-flash 5 L, 60 A, 40 μm; Yamazen Science, Burlingame, CA) using step-gradient elution (v/v hexane: ethyl acetate = 90:10, 30 min; 60:40, 23 min; 40:60, 1 min; 0:100, 16 min; flow rate: 35 ml/min, detection: 280 nm). The peak from 50 to 70 min was collected and evaporated to dryness. All collected samples were analyzed by LC–MS and GC–MS.

This method was then scaled up to the prepared assay sample. By connecting the injection column with silica gel (60 A, 70 μm) hand-packed in an empty column (5 L) with a high-performance flash column, the soluble fraction was separated using step-gradient elution (v/v Hexane: ethyl acetate = 90:10, 130 min; 60:40, 45 min; 40:60, 50 min; 0:100, 50 min; flow rate, 40 ml/min; detection, 280 nm). The peaks from 175 to 225 min were collected and evaporated to dryness. The same fractionation was performed multiple times to obtain a fraction of ca. 100 g from cacao nibs (55 kg) and was used in experiments with *D. melanogaster*.

### Mass spectrometry

The cacao extracts were analyzed by A 1290 Series LC System (Agilent Technologies, Santa Clara, CA, USA), using an ACQUITY UPLC BEH C8, 1.7 μ m, 2.1 × 50 mm column (Waters Manchester, USA). The mobile phase was water containing 0.1% formic acid (solvent A) and acetonitrile containing 0.1% formic acid (solvent B). The flow was set at 0.3 mL/min, and the column temperature was 40 °C. The gradient profile was 0 min, 80% B; from 0 to 5 min, linear gradient from 80 to 100% B; from 5 to 8 min, isocratic 100% solvent B. LC-Mass spectrometry was performed using a 6530 Accurate-Mass Q-TOF (Agilent Technologies, Santa Clara, CA, USA) instrument equipped with an electrospray ion source in both negative and positive ionization mode. Both the drying gas (280 °C, 10 L/min) and nebulizer gas flow (50 L/min) were nitrogen. The sheath gas (7.5 L/min, 350 °C) was also nitrogen. The fragmentor voltage was 100 V in both positive and negative mode. Mass spectra were acquired over the range *m/z* 90‒1300.

GC-mass spectrometry was performed using a GC–MS TQ8040 system (Shimadzu Co., Kyoto, Japan). Analyses of cacao extract were performed using a DB-5HT (Agilent) capillary column (30 m × 0.25 mm I.D., 0.1 μm film thickness) with helium as the carrier gas. Sample injection was performed in split mode (1/10). The oven temperature was increased from 100 °C (1 min hold) to 400 °C at a rate of 30 °C/min and then to 400 °C (2 min hold) The mass spectrometer was operated in the electron ionization mode. The analyses were then recorded in total ion count (TIC) mode. The mass spectra were acquired over the range *m/z* 35‒600.

### SIRT1 deacetylase assays by CycLex

The human SIRT1 deacetylase assays were performed using a CycLex SIRT1/Sir2 Deacetylase Fluorometric Assay Kit (CycLex, Columbia, MD, USA) according to the manufacturer’s instructions. In brief, the deacetylase reactions were carried out with recombinant SIRT1 and with various concentrations of NAD+ (12.5–200 μM) or fluoro-substrate peptide (0.5–20 μM) at room temperature. The fluorescence intensities were measured for 60 min at 1-min intervals using a microtiter plate fluorometer with excitation at 350 nm and emission at 450 nm.

### Histone deacetylation assays

Human monocyte THP-1 (ATCC TIB-202; purchased from ATCC) cells were grown in RPMI-1640 medium supplemented with 10% fetal bovine serum (FBS), 2 mM l-glutamine, and 50 μg/mL penicillin–streptomycin at 37 °C in a humidified chamber containing 95% air and 5% CO_2_. Semi-confluent cells were seeded at 1 × 10^7^ cells/well and then exposed to 100 nM trichostatin A (TSA) and 10 or 30 μM of either Fa-Trp21 or Fa-Trp23 for 24 h. Acetylated histone was extracted with an EpiQuik Total Histone Extraction Kit (EpiGentek, Farmingdale, NY) according to the manufacturer’s instructions. The proportion of acetylated histone in the total extracted histone was measured with an EpiQuik Total Histone Acetylation Detection Fast Kit (EpiGentek) according to the manufacturer’s instructions.

### Fatty cell culture model

HepG2 (ATCC HB-8065; purchased from ATCC) cells were maintained in Dulbecco’s modified Eagle’s medium (DMEM) supplemented with 10% (v/v) FBS, 10,000 U/mL penicillin and 10 mg/mL streptomycin. Cells were sub-cultured every 48–72 h at a confluency of 80–90%. The effect of palmitate^39,40^ was examined at 5 × 10^5^ cells per well. After confluency, HepG2 cells were exposed to Fa-Trp with or without 300 μmol/L fatty acid (palmitic acid) conjugated by bovine serum albumin as an inducer of steatosis for 24 h. The number of cells positive for Oil Red O staining was measured by taking a microscopic image of the cell culture and measuring the number of cells stained red per 40 mm^2^.

Mouse 3T3-L1 (ATCC CL-173; purchased from ATCC) cells were cultured in DMEM containing 10% fetal bovine serum at 37 °C in a 5% CO_2_ incubator. 3T3-L1 cells were maintained on culture plates. After confluency, the cells were treated for 48 h with a hormone mixture containing 10 μg/mL insulin, 0.5 μM dexamethasone, and 0.5 mM IBMX (Sigma-Aldrich, MO, USA), and then exchanged with DMEM containing insulin. The cells were differentiated into adipocytes until day 8. After 24 h of stimulation with a reagent such as Fa-Trp, Oil Red O staining was performed. Culture media were changed every 2 days. During treatment, cells were treated with different concentrations of Fa-Trp for 6–8 days before further analysis. After removing the staining solution, the dye retained in the cells was eluted into isopropanol and OD 540 nm was determined.

### Fly stocks, maintenance, and experiments

A *w*^*1118*^ line (stock number: 108479; identical to Iso31, isogenic *w*^*1118*^ stock in Bloomington Drosophila Stock Center #5905) was obtained from the Drosophila Genetic Resource Center (Kyoto, Japan) and used as the wild-type experimental animal.

Flies were raised under a 12-h light–dark cycle at 25 °C and 50% humidity on standard food (SF) consisting of 10% (w/v) glucose, 7% (w/v) cornmeal, 4% (w/v) yeast extract, and 0.55% (w/v) agar medium containing 0.3% (v/v) propionic acid and 0.35% (v/v) butyl p-hydroxybenzoate as antifungal agents, in keeping with our previous studies^41^. We performed all studies using repeated observation and measurements at the same time each day whenever possible.

The Fa-Trp21, Fa-Trp23, and resveratrol (Res) added to the feed was commercially acquired. These compounds were dissolved in 100% ethanol to final concentrations of 700 μM (w/v) (for D-Trp and T-Trp) or 100 μM (w/v) (for Res). An equal amount of ethanol was added to SF.

A longevity assay was performed as described in our previous study^41^. Briefly, in each lifespan experiment, newly eclosed adult flies (within 12 h of eclosion) of each treated group were collected, anesthetized with diethyl ether, and separated into virgin males and females. Male flies (n ≤ 20) were placed in a single vial (diameter, 28 mm; length, 100 mm) containing SF, D-Trp, T-Trp, or Res and transferred every 3–4 days to a fresh vial. The number of dead flies was recorded until no living flies remained. A modified version of the CAFE assay described by Ja et al.^42^ was used to quantify feed consumption. Liquid feed was loaded on a 5 µl glass capillary and inserted into a 50 ml conical tube. The tube contained adult male flies, which were allowed to feed for a fixed time (approximately 20 h). The quantity that was consumed was measured.

The Hill climbing assay was performed by the following method. Within 12 h of eclosion, virgin male adults were bred with SF and SF containing cacao-derived tryptamine. After 4, 12, and 20 days, only one animal was taken out of the breeding vial and sealed in a 10 mL thin volumetric cylinder. After tapping the vial to cause the fly to fall to the bottom, we measured how far it climbed at a given time.

In the study to investigate gene expression levels, within 12 h of eclosion, virgin adults were first separated into three groups by sex, bred in SF, D-Trp, and T-Trp for 12 days, and total RNA was extracted using three samples and RNeasy mini Kit (Qiagen, Hilden, Germany) in units of ten animals each. The sequencing library construction and the RNA-seq were performed by Macrogen Japan Corp. (Kyoto, Japan) using the NovaSeq 6000 sequencer (Illumina, San Diego). Then, fastq files with a paired-end 101 bp were generated. Adaptor sequences and low-quality bases were removed using Cutadapt 3.3. The filtered reads were mapped to the *D. melanogaster* reference genome (Ensembl BDGP6.32) using STAR 2.7.5a. The gene and isoform expression levels were quantified as expected counts, transcripts per million and fragments per kilobase of exon per million mapped reads using RSEM 1.3.3. For differential gene expression analysis, expected counts were used as the gene expression level. Genes with expected count values of zero in all samples were removed. A differential gene expression analysis was conducted using edgeR 3.32.1.

Gene pathway information was obtained from KEGG https://www.genome.jp/kegg/pathway.html). For the enrichment and gene set analysis, the Parametric Analysis of Gene-set Enrichment (PAGE) was conducted using fold change values for each gene between two experimental groups^43^.

### Statistical analysis

Data on in vitro experiments were shown by the average value of three independent test results ± S.E. (n = 3). A significant difference test was performed by Dunnett's method using GraphPad PRISM Version5 (GraphPad Software, San Diego, CA, USA). The survival rate of the fly was analysed using the Kaplan–Meier method with Wilcoxon signed-rank test. A p value < 0.05 was considered statistically significant.

## Supplementary Information

Supplementary Information 1.

Supplementary Information 2.

Supplementary Information 3.

Supplementary Information 4.

Supplementary Information 5.

Supplementary Information 6.

Supplementary Information 7.

Supplementary Information 8.

Supplementary Information 9.

Supplementary Information 10.

## Data Availability

All data generated or analysed during this study are included in this published article and its supplementary information files.
