# Supplementary material for: Fatty acid tryptamide from cacao elongates Drosophila melanogaster lifespan with sirtuin-dependent heat shock protein expression
Source: Sci Rep. 2022 Jul 15;12:12080. doi: 10.1038/s41598-022-16471-1 (PMC9287426; doi:10.1038/s41598-022-16471-1)
Supplement: Supplementary file 1 — Supplementary Information 1. [file 41598_2022_16471_MOESM1_ESM.pdf]

Experiment#1

Plate#1

	1	2	3	4	5	6	7	8	9	10	11	12
A	0.000	0.000	0.000	0.000	0.000	0.000	0.000	0.000	0.000	0.000	0.000	0.000
B	0.000	0.000	0.703	0.499	0.417	0.327	0.696	0.535	0.493	0.452	0.000	0.000
C	0.000	0.000	0.619	0.618	0.519	0.437	0.635	0.558	0.540	0.490	0.000	0.000
D	0.000	0.000	0.788	0.376	0.335	0.304	0.757	0.468	0.377	0.287	0.000	0.000
E	0.000	0.000	0.701	0.716	0.650	0.520	0.688	0.755	0.616	0.542	0.000	0.000
F	0.000	0.000	0.652	0.799	0.680	0.620	0.650	0.476	0.677	0.574	0.000	0.000
G	0.000	0.000	0.749	0.567	0.551	0.445	0.726	0.840	0.570	0.503	0.000	0.000
H	0.000	0.000	0.000	0.000	0.000	0.000	0.000	0.000	0.000	0.000	0.000	0.000

Endpoint

Lm1 450

Automix: Off
Calibrate: Off

Imported Data

Wavelength Combination: !Lm1

Data Type: Absorbance

Unknown
