# Supplementary figures and images for: Fatty acid tryptamide from cacao elongates Drosophila melanogaster lifespan with sirtuin-dependent heat shock protein expression
Source: Sci Rep. 2022 Jul 15;12:12080. doi: 10.1038/s41598-022-16471-1 (PMC9287426; doi:10.1038/s41598-022-16471-1)

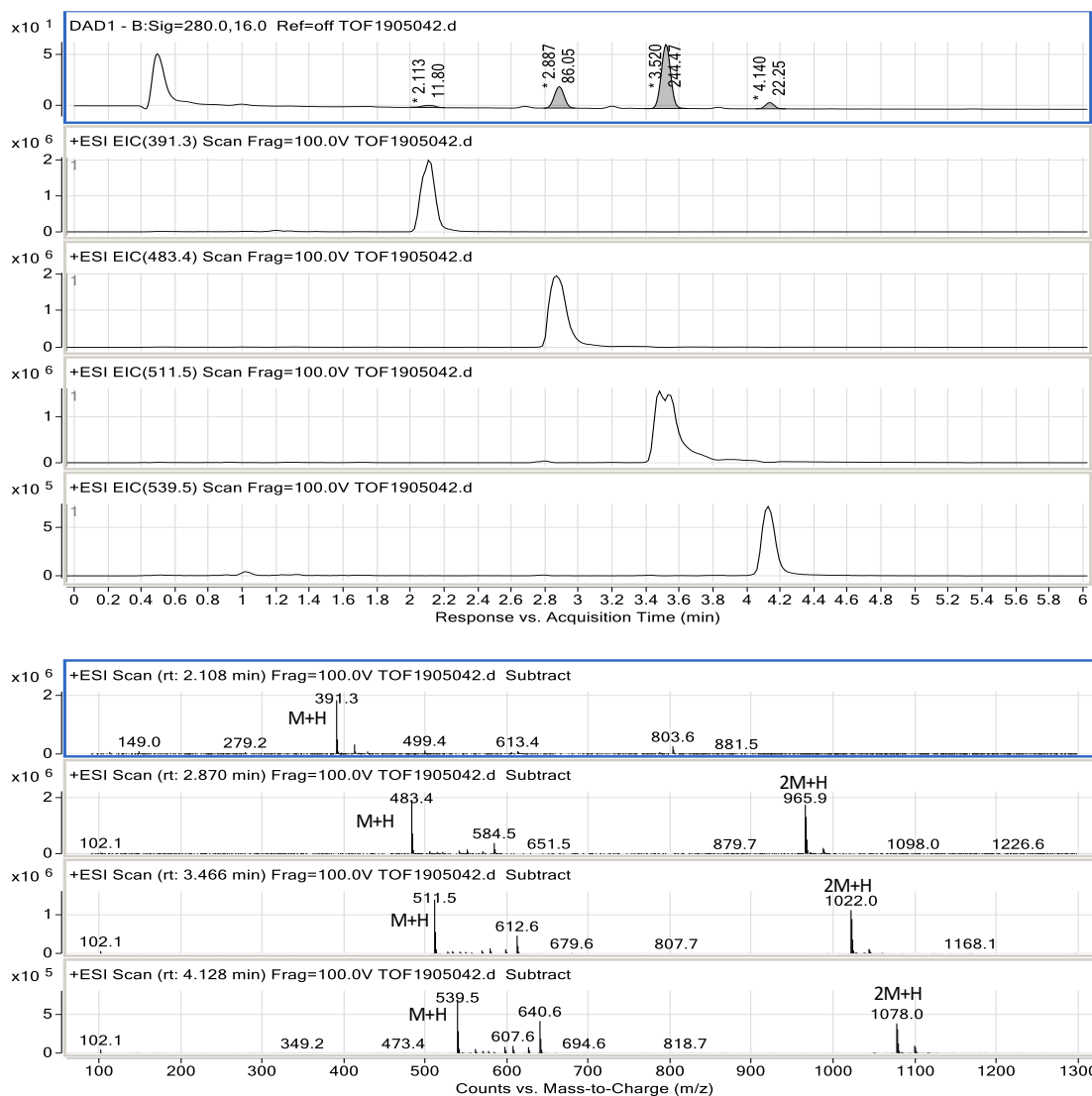

LC/MS result of the from 50 to 70 min fraction

Supplement: Supplementary file 8 — Supplementary Information 8. [file 41598_2022_16471_MOESM8_ESM.pdf]
